# Lemmel’s Syndrome: A Rare Cause of Obstructive Jaundice Due to Periampullary Duodenal Diverticulum

**DOI:** 10.7759/cureus.95981

**Published:** 2025-11-03

**Authors:** Oualid Hadadia, Hanane Aksim, Intissar Lasfar, Meryem Belhamdiya, Rachid Akka

**Affiliations:** 1 Gastroenterology and Hepatology, Moulay Hassan Hospital Guelmim, Guelmim, MAR; 2 Hepatogastroenterology, Avicenne Military Hospital, Marrakech, MAR; 3 Gastroenterology, Avicenne Military Hospital, Marrakech, MAR; 4 Gastroenterology, Avicenne Military Hospital, Cadi Ayyad University, Marrakech, MAR

**Keywords:** bile duct, biliary obstruction, case report, endoscopic retrograde cholangiopancreatography, endoscopic sphincterotomy, lemmel’s syndrome, minimally invasive, obstructive jaundice, periampullary diverticulum, retrograde cholangiopancreatography

## Abstract

Lemmel’s syndrome is a rare cause of obstructive jaundice resulting from extrinsic compression of the distal common bile duct by a periampullary duodenal diverticulum. Its clinical presentation often mimics more common etiologies, such as choledocholithiasis or malignancy, leading to frequent underdiagnosis. We report a case of a 72-year-old man who presented with progressive jaundice, dark urine, and mild right upper quadrant discomfort. Laboratory tests revealed elevated total bilirubin and cholestatic liver enzymes, with normal inflammatory markers. Imaging studies, including abdominal ultrasound, contrast-enhanced computed tomography, and magnetic resonance cholangiopancreatography (MRCP), demonstrated biliary dilatation and a gas- and fluid-filled periampullary diverticulum compressing the distal common bile duct, without evidence of stones or masses. Endoscopic retrograde cholangiopancreatography (ERCP) confirmed the diagnosis and allowed therapeutic intervention through sphincterotomy and temporary biliary stenting. The patient’s jaundice resolved completely, liver function tests normalized, and he remained asymptomatic during a six-month follow-up period. Lemmel’s syndrome should be considered in elderly patients presenting with obstructive jaundice in the absence of choledocholithiasis or malignancy. Prompt recognition through appropriate imaging and endoscopic management ensures excellent outcomes and prevents unnecessary surgical procedures.

## Introduction

Periampullary duodenal diverticula (PDD) are mucosal outpouchings located near the ampulla of Vater, incidentally detected in approximately 10-20% of patients undergoing endoscopic or radiological examinations [1]. Most PDDs are asymptomatic; however, in some cases, they can lead to biliary or pancreatic complications, such as obstructive jaundice, pancreatitis, or cholangitis [2]. This clinical condition is known as Lemmel’s syndrome, first described by Lemmel in 1934 [3]. Due to its rarity and nonspecific clinical presentation, Lemmel’s syndrome often remains underdiagnosed, potentially resulting in unnecessary surgical or invasive procedures [4]. Here, we report a case of obstructive jaundice secondary to a periampullary diverticulum, successfully managed by endoscopic intervention.

## Case presentation

A 72-year-old man presented with progressive jaundice, mild right upper quadrant discomfort, and dark urine for 10 days. He denied fever, chills, or weight loss. His past medical history was unremarkable, and he was not taking any hepatotoxic medications. On physical examination, he was alert, afebrile, and hemodynamically stable. Scleral icterus was evident, and mild tenderness was elicited in the right upper quadrant without palpable hepatomegaly or masses. No stigmata of chronic liver disease were observed.

Laboratory evaluation revealed a markedly elevated total bilirubin level of 26.8 mg/dL, predominantly conjugated (direct bilirubin = 21.5 mg/dL). Alkaline phosphatase (ALP) and gamma-glutamyl transferase (GGT) levels were also significantly increased (680 U/L and 520 U/L, respectively), consistent with a cholestatic pattern of liver injury. Table 1 presents the patient’s biochemical values at admission.

**Table 1:** Biochemical laboratory values of the patient.

Parameter	Patient value	Reference range	Interpretation
Total bilirubin	26.8 mg/dL	0.2 – 1.2 mg/dL	Markedly elevated
Direct (conjugated) bilirubin	21.5 mg/dL	<0.3 mg/dL	Predominantly conjugated
Aspartate aminotransferase (AST)	95 U/L	<40 U/L	Mildly elevated
Alanine aminotransferase (ALT)	88 U/L	<41 U/L	Mildly elevated
Alkaline phosphatase (ALP)	680 U/L	40 – 129 U/L	Markedly elevated
Gamma-glutamyl transferase (GGT)	520 U/L	8 – 61 U/L	Markedly elevated
Albumin	3.6 g/dL	3.5 – 5.0 g/dL	Normal
Prothrombin time (PT)	14.8 s	11 – 14 s	Slightly prolonged
C-reactive protein (CRP)	3 mg/L	<5 mg/L	Normal

Ultrasound demonstrated intrahepatic and extrahepatic bile duct dilatation but no gallstones. Contrast-enhanced computed tomography revealed a fluid- and gas-filled outpouching adjacent to the ampulla compressing the distal common bile duct. Magnetic resonance cholangiopancreatography (MRCP) confirmed extrinsic compression of the common bile duct by a periampullary diverticulum without stones or masses.

**Figure 1:**
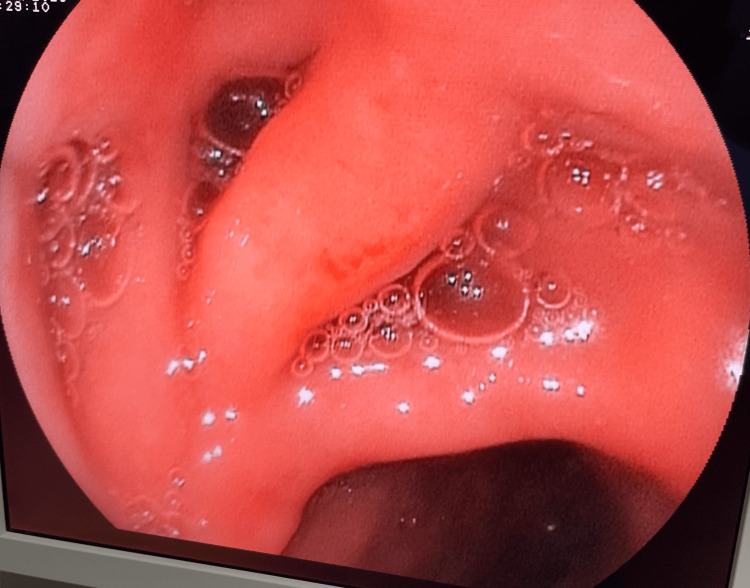
Endoscopic image showing a periampullary duodenal diverticulum adjacent to the duodenal lumen.

Endoscopic retrograde cholangiopancreatography (ERCP) identified the diverticulum near the papilla with evidence of bile duct compression (Figure 1).

The main differential diagnoses considered included choledocholithiasis, cholangiocarcinoma, pancreatic head carcinoma, and ampullary tumors, all of which can present with painless obstructive jaundice and biliary dilatation. However, the absence of biliary stones, mass lesions, or mural thickening on imaging, combined with the demonstration of a periampullary diverticulum exerting direct extrinsic compression on the distal common bile duct, favored the diagnosis of Lemmel’s syndrome.

**Figure 2:**
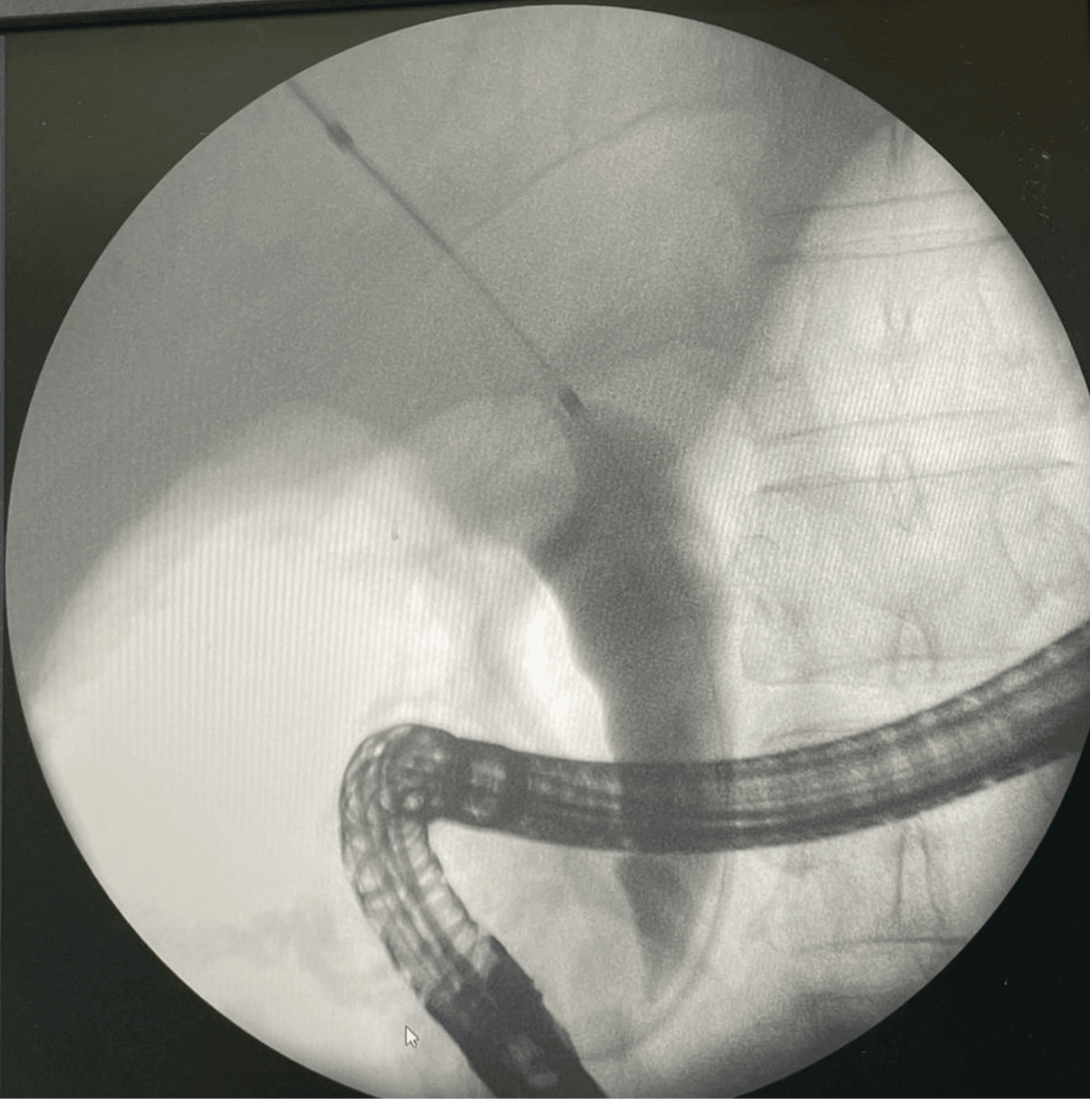
Cholangiogram showing biliary ductal dilatation.

**Figure 3:**
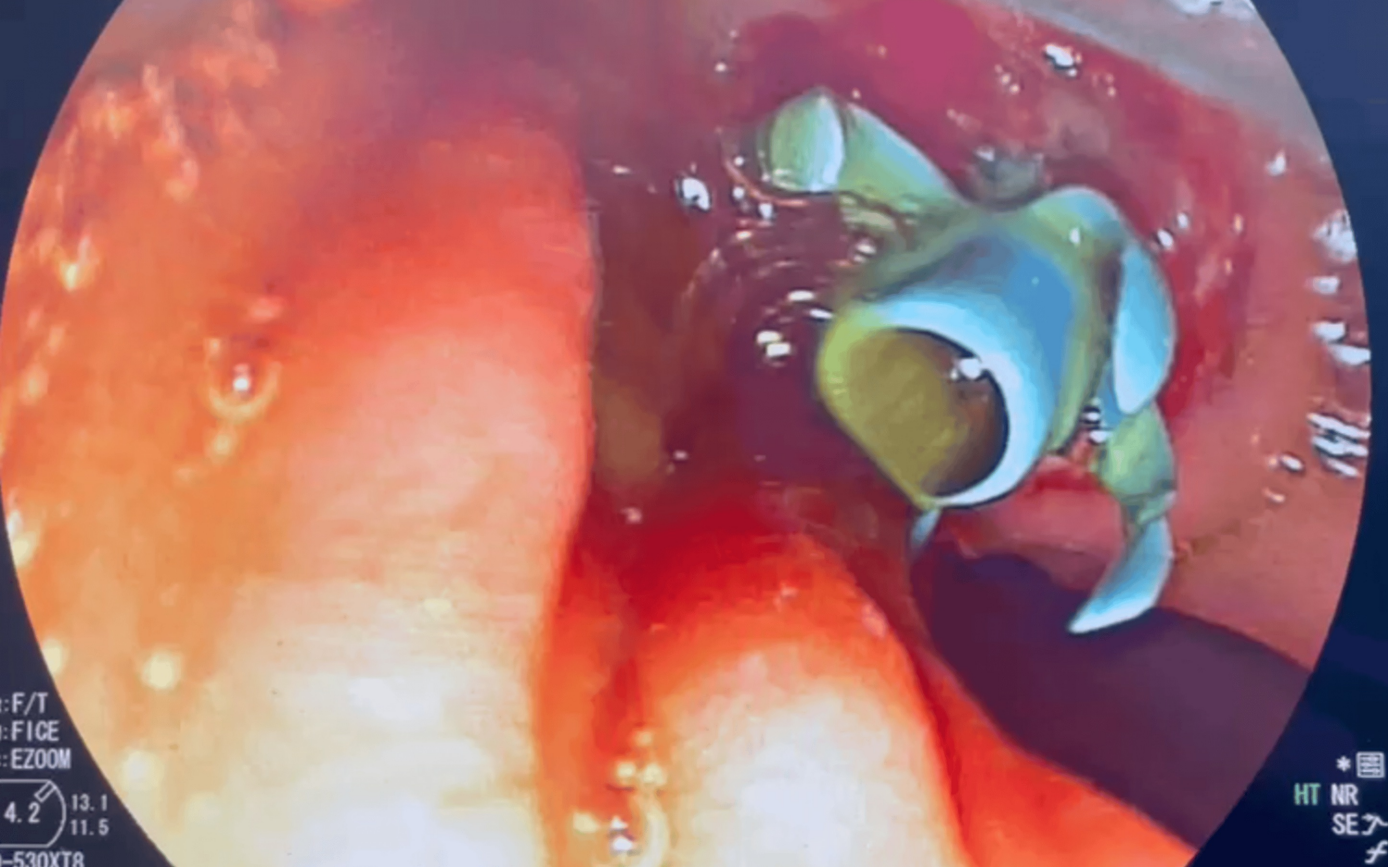
Endoscopic retrograde cholangiopancreatography image showing biliary stent placement after endoscopic sphincterotomy with restoration of bile flow.

Cholangiogram obtained during ERCP showing biliary ductal dilatation secondary to extrinsic compression of the distal common bile duct by the diverticulum (Figure 2). Endoscopic sphincterotomy and temporary biliary stenting were performed (Figure 3).

After endoscopic placement of a biliary stent, the patient’s clinical condition improved rapidly, with progressive resolution of jaundice and pruritus within two weeks. Liver function tests demonstrated a steady normalization pattern. The stent was removed after six weeks, and subsequent follow-up over three months showed complete clinical recovery and normalization of hepatic parameters. Follow-up ultrasonography revealed a normal bile duct diameter (5 mm) with no residual dilatation or periampullary diverticulum compression (Table 2).

**Table 2:** Evolution of laboratory parameters.

Parameter	On admission	3 weeks after stent placement	3 months after stent removal	Reference range
Total bilirubin (mg/dL)	26.8	3.5	0.9	0.2 – 1.2
Direct bilirubin (mg/dL)	21.5	2.4	0.3	<0.3
Aspartate aminotransferase (U/L)	95	45	32	<40
Alanine aminotransferase (U/L)	88	48	35	<41
Alkaline phosphatase (U/L)	680	160	115	40 – 129
Gamma-glutamyl transferase (U/L)	520	130	60	8 – 61
Albumin (g/dL)	3.6	3.8	3.9	3.5 – 5.0
Prothrombin time (s)	14.8	13.5	13.2	11 – 14
C-reactive protein (mg/L)	3	2	1	<5

## Discussion

PDD are mucosal outpouchings located near the ampulla of Vater, typically within 2-3 cm of the papilla. These diverticula are relatively common, found in approximately 10-20% of individuals undergoing endoscopy or imaging, but the vast majority remain asymptomatic. In rare cases, they may cause biliary or pancreatic complications such as cholangitis, pancreatitis, or obstructive jaundice, a clinical presentation known as Lemmel’s syndrome [3,5].

The pathophysiology of Lemmel’s syndrome is multifactorial. The periampullary diverticulum can exert mechanical compression on the distal common bile duct or cause functional obstruction by affecting the sphincter of Oddi's motility through chronic inflammation or fibrosis. Furthermore, inflammation of the diverticulum itself can worsen the obstruction [6,7]. These mechanisms highlight why this syndrome can mimic more common causes of biliary obstruction, such as choledocholithiasis or malignancy.

Diagnosing Lemmel’s syndrome is often challenging due to its nonspecific clinical features and similarity to other causes of obstructive jaundice. Ultrasound may reveal bile duct dilatation but typically fails to identify the diverticulum. Advanced imaging modalities such as CT and MRCP offer better visualization, enabling detection of a gas- or fluid-filled diverticulum compressing the common bile duct [8]. MRCP, in particular, is advantageous for noninvasive assessment of both biliary and pancreatic ducts and can delineate the extrinsic nature of the obstruction [9].

ERCP remains the gold standard for both diagnosis and treatment, allowing confirmation of the diverticulum's relationship to the biliary system and facilitating therapeutic interventions, such as sphincterotomy or stent placement, to relieve obstruction [10]. Endoscopic management has been shown to provide excellent outcomes in most patients, avoiding the need for surgery unless complications or refractory symptoms arise.

In summary, clinicians and radiologists should maintain a high index of suspicion for Lemmel’s syndrome in elderly patients presenting with obstructive jaundice but no gallstones or malignancy. Early and accurate diagnosis through appropriate imaging and endoscopic intervention can prevent unnecessary surgical procedures and ensure prompt symptom resolution.

## Conclusions

Lemmel’s syndrome is a rare but important cause of obstructive jaundice resulting from extrinsic compression of the common bile duct by a periampullary duodenal diverticulum. Its nonspecific presentation makes it a diagnostic challenge. Recognition through advanced imaging and confirmation by ERCP allows for effective endoscopic treatment, sparing patients from unnecessary surgery. Awareness of this syndrome should be increased among clinicians to improve patient outcomes.

In our reported case, endoscopic sphincterotomy and temporary biliary stenting effectively relieved the obstruction caused by the periampullary diverticulum. The patient’s jaundice resolved quickly, liver function normalized, and no recurrence was observed at six months. This underscores the value of minimally invasive endoscopic management in Lemmel’s syndrome and supports its role as first-line therapy.
